# Financial incentives to stop smoking: Potential financial consequences of different reward schedules

**DOI:** 10.18332/tpc/190617

**Published:** 2024-07-12

**Authors:** Gintare Valentelyte, Aishling Sheridan, Paul Kavanagh, Frank Doyle, Jan Sorensen

**Affiliations:** 1 Converge: Centre for Chronic disease and Population Health Research, School of Population Health, RCSI University of Medicine and Health Sciences, Dublin, Ireland; 2 Healthcare Outcome Research Centre, School of Population Health, RCSI University of Medicine and Health Sciences, Dublin, Ireland; 3 Tobacco Free Ireland Programme, Health Services Executive (HSE), Ireland; 4 Department of Epidemiology and Public Health, School of Population Health, RCSI University of Medicine and Health Sciences, Dublin, Ireland; 5 Department of Health Psychology, School of Population Health, RCSI University of Medicine and Health Sciences, Dublin, Ireland

**Keywords:** Ireland, cost-effectiveness, financial incentives to stop smoking, FISS, smoking intervention, budget impact

## Abstract

**INTRODUCTION:**

Financial incentives to stop smoking (FISS) programs have been implemented internationally to encourage people who smoke to quit smoking. However, such programs require that the financial reward structure and its resulting effects on smoking quit rates are considered. We analyzed a number of scenarios for FISS reward schedules for current smoking individuals in Ireland, with a view to identify the potential implications in terms of financial consequences and expected effects.

**METHODS:**

Using national QuitManager services data 2021–2023, we defined smoking quit rates for smokers currently using the national Health Services Executive stop smoking services in Ireland. Smoking quit rates at 4, 12 and 52 weeks were defined, and additionally defined by sex, age and education level. Using scenarios assuming different FISS reward sizes, structures and targeted population sub-groups, we estimated the number of additional quitters, budget impact, and incremental cost-effectiveness ratio.

**RESULTS:**

A FISS program, if implemented for a cohort of 3500 smokers can result in a budget impact ranging €250000 – €870000. The cost-effectiveness trade-off between different payment schedules and the expected effect size suggested that FISS are cost-effective even at a moderate effect size. A FISS program implemented to approximately 20000 smokers nationally would cost between €2.0 million and €4.8 million, subject to the chosen reward schedule. Across social groups, FISS is more cost-effective for females, individuals in the youngest age group, and individuals with a medium level of education.

**CONCLUSIONS:**

This analysis highlights the importance of considering different FISS schedules and potential quit effects, when designing such programs. We highlight that FISS programs should be targeted at certain social groups to achieve highest long-term smoking cessation rates. We also identified important challenges that decision-makers face when designing the reward structure of FISS programs. The acceptability or otherwise of the FISS structures may differ among stakeholders and should be explored.

## INTRODUCTION

Programs with financial incentives to stop smoking (FISS) have been implemented internationally as a way to encourage smokers to quit^1^. When introducing such programs, it is necessary to consider how the financial rewards should be arranged, i.e. when, how frequently and how much participants should be rewarded^2^. The incentive structure and the amount of the financial reward are likely to influence the program’s effectiveness in terms of smokers’ willingness to participate and their long-term success in quitting smoking^3^.

Limited research offers insights into these important aspects. A Cochrane systematic review and meta-analysis of existing published evidence of the effects of FISS from 2019 found that FISS is effective in supporting smoking cessation^1^. The review suggested a substantial FISS effect size of 1.49 (95% CI: 1.28–1.73), suggesting that financial incentives are 50% more likely to lead to smoking cessation than other types of support^1^. The review was based on evidence from a range of randomized controlled trials, which varied substantially in their reporting of the FISS structures. The majority of FISS were implemented in the form of contingent rewards (for smoking abstinence), with higher incentive rewards for long-term abstinence, and in addition to existing and currently delivered smoking cessation interventions, e.g. Cognitive Behavioral Therapy^1^. The financial incentive amounts also varied, ranging from zero (relying on individuals self-depositing their own money and accessing once smoking cessation is proven) to amounts from US$45 to US$1185 per smoker entering the program^1^. However, the meta-analysis could not identify a statistically significant relationship between the size of the financial reward and successful quit rate, in contrast to some trials reporting a linear relationship with higher financial incentive rewards associated with higher cessation rates^1^. A more recent systematic review of the relationship between reward amounts and their effect on smoking quit rates concluded that higher rewards are only weakly associated with greater smoking cessation rates^4^. The authors highlight some of the difficulties in evaluating the relationship between reward schedules and smoking cessation rates and, in particular, the variation among different groups of smokers. Judging from these reviews, it remains unclear which FISS reward schedules could be better at achieving high quit rates and if an ‘optimal’ reward schedule can be established or recommended^1,4^.

Theoretically, we expect that willingness to engage with the smoking cessation support program will increase when a FISS reward system is introduced^5-7^. Financial incentives may function in accordance with operant conditioning behavioral processes (positively rewarding the desired behavior) or by offering a short-term benefit for changing behavior that eventually produces a long-term benefit but is viewed as less immediate to the individual (delay discounting)^5-7^. Thus, the marginal utility of individuals today is correlated with historical consumption; changes today may lead to a small short-term effect but increasingly large long-term effects^5^. We also expect that the willingness to engage will increase with higher financial rewards. Although these relationships are unlikely to be proportional, we simplify the analysis by assuming linear relationships for convenience.

It is highly likely that the relationship will be different for different groups of smokers defined by sociodemographic variables^8^. Socioeconomic status has been hypothesized to directly influence environmental and psychosocial variables, which in turn directly impact health behaviour^8^. For example, smoking individuals with high disposable income may be less likely to engage in a smoking cessation program than those with low disposable income (i.e. a lower amount of income available for other expenses after tax deductions), as the financial reward will be more attractive for people with lower disposable income. Smoking individuals identified by other sociodemographic characteristics such as sex, age, education level and deprivation, and smoking habits such as regular or occasional consumption of tobacco products may have different willingness to engage in the smoking cessation program and have a different rate of successful smoking cessation after 12 months, due to social factors more influential within their peer groups (e.g. the number of friends and family who smoke).

Ample evidence has documented the positive health consequences of smoking cessation^9^ and these may be part of the general incentives to quit smoking. Participants in smoking cessation programs with financial incentives will experience both health and financial benefits from successful smoking cessation^9^. Substantial financial benefits will also arise from saved expenditures to purchasing tobacco products. We therefore expect these incentives to increase if additional financial rewards for smoking cessation are introduced.

Given the mixed evidence reported to date^1,4^, it is important to explore which FISS reward schedules could potentially achieve the highest smoking quit rates. We provide an analysis of a number of scenarios for FISS reward schedules with a view to identifying the potential implications in terms of financial consequences and expected effects. We demonstrate these consequences in terms of the number of additional quitters, the budget impact of the program, and the cost-effectiveness expressed as incremental cost per successful 12-month quitter. This analysis is aimed at informing and supporting decision-makers who are responsible for designing financial smoking cessation programs. Specifically, we:

1. Assess the consequences of different incentive reward schedules of a likely implementation scenario specified by the Health Services Executive;
2. Illustrate the cost-effectiveness of different reward schedules and effect sizes;
3. Assess the potential budget impact of different reward schedules; and
4. Consider the consequences of the FISS program across different sociodemographic characteristics.

## METHODS

To explore the effect on quit rates of various FISS reward schedules, we obtained national data from the QuitManager service for 2021–2023. Since 2018, the QuitManager data have been used to monitor and evaluate care quality and improvement across smoking cessation services delivered across Ireland^10^. The QuitManager data capture the smoking quit rates and outcomes of all smoking individuals in Ireland who have used the Health Services Executive (HSE) stop smoking support program, in line with the Russell Standards for smoking cessation^10,11^. The stop smoking support services consist of multiple behavioral support sessions with an advisor during which a smoking quit date is set, individuals receive care and support and are followed up over 12 months^10^. We used these data to establish baseline smoking quit rates at 4, 12, and 52 weeks for individuals who signed up and committed to following the program. The quit rates reported at each time frame are based on both self-reported and biochemical verification tests for smoking abstinence^12,13^. We calculated the smoking quit rates by sex (male, female), age (18–49, 50–59, ≥60 years), and education level (low – less than primary or primary level; medium – secondary level; high – tertiary level or higher) (Supplementary file).

Based on these data, we calibrated a relatively simple mathematical model, which estimated the number of additional quitters, the budget impact, and the incremental cost-effectiveness ratio defined as cost per additional quitter. The model was applied to a range of effect sizes retrieved from the literature^1,14,15^, defined as the additional proportion of smokers who are successful in quitting smoking after 12 months. Given the uncertain nature of the relationship between the financial incentive size and its effect size as per the recent Cochrane review^1^, we assumed a linear relationship between the effect and reward sizes in our analysis, i.e. greater smoking quit success rate with higher incentive reward. For parsimony, this relationship – which may not be true in practice – was used to highlight the possible outcomes when considering different financial incentive structures. This general framework was applied in four specific analyses.

### Consequences of different FISS reward schedules for HSE-specified scenarios

We have specified three different scenarios for the FISS reward structure, as shown in Table 1. The FISS reward size of €400 was adapted to the Irish context based on a recent UK study^16^ and identified as appropriate by the HSE. To compare with the different models, we explored the consequences of a scenario specified by the HSE. This scenario assumed that the FISS program would be implemented across Sláintecare Healthy Community Program areas (SHCPAs) in a targeted cohort of 3500 current HSE service users, representing approximately 18% of total service activity. By nature, SHCPAs have adverse area-based deprivation scores^17^. Therefore, it was assumed that the participants from these areas have a low socioeconomic background, e.g. low education level. Based on QuitManager data for 2022–2023 specific to services delivered in SHCPAs, we assumed that 68.1% of this cohort had entered the FISS program. Additionally, from these data, we defined the smoking quit rates for this group as follows: 4-week quit rates – 53.6%; 12-week quit rates – 36.8%; 52-week quit rates – 16.8%. We report the consequences of each payment schedule (Table 1), with the effect sizes ranging from 5% to 60% of the base-case quit rate^1,14,15^.

**Table 1:** Summary of the scenarios of FISS reward schedules used in modelling

*Reward schedule*	*Scenario 1 (€)*	*Scenario 2 (€)*	*Scenario 3 (€)*
Enrolment	50	0	100
4-week quit	50	0	0
12-week quit	100	0	0
52-week quit	200	400	300
Total	400	400	400

FISS reward schedules adapted from reviewed studies^1^. Scenario 1: reward paid at enrolment, and at 4, 12 and 52 weeks for individuals who have successfully abstained from smoking (successful quit)^16^. Scenario 2: reward paid only at 52 weeks for successful quit. Scenario 3: reward paid at enrolment and at 52 weeks for successful quit. The FISS reward size of €400 was adapted to the Irish context from a previous study^16^.

We assumed that the financial rewards should be paid to all participants. This implies a substantial additional cost burden for the smoking cessation program. When deriving the cost-effectiveness, the reward system assumed that the added cost burden applies for the full cohort of participants (the incremental costs) in relation to the increased number of quitters, which is determined based on the assumed effect size (the incremental effect).

### Consequences on cost-effectiveness for different incentive payment schedules and effect size

For each of the three specified reward schedules, we explored the cost-effectiveness defined as cost per successful quitter. We explored the potential impacts in terms of achieving the highest quit rates and the expected FISS effect size. Based on the national level QuitManager data for 2021–2022 of all HSE service users, we defined the base-case smoking quit rates as follows: 4-week quit rates – 53.6%; 12-week quit rates – 36.8%; 52-week quit rates – 16.8%.

### Consequences on the budget impact of FISS at the population level

We considered the budget impact of the different reward schedules if they were offered at a national level to the whole smoking population in Ireland. There are approximately 0.8 million smoking individuals in Ireland, 50% of whom indicated that they have made an attempt to quit smoking within the last 12 months^18^. Of those who have indicated that they have made a quit attempt, approximately 20000 have used support from the HSE stop smoking program in the form of face-to-face and/or telephone Quitline services^19,20^. We assessed the budget impact of potentially implementing the FISS program, assuming the additional FISS effect of quitting is 50%, as per the overall effect reported in a recent Cochrane review^1^.

### Consequences of FISS by sociodemographic characteristics

To capture the consequences of FISS across sociodemographic characteristics, we analyzed quit rates by sex, age, and education level using the available QuitManager data. We used Scenario 3 to define the FISS reward schedule and assumed that effect sizes ranged from 5% to 60%^1,14,15^. We defined the base-case smoking quit rates at 4, 12, and 52 weeks using the national level QuitManager data for 2021–2022 of all HSE service users. We also imposed these on a cohort of 3500 smokers enrolled in the FISS program in SHCPAs.

We reported the consequences in terms of the number of additional quitters, the budget impact, and the incremental cost-effectiveness ratio (ICER), i.e. cost per successful quitter. We compared the cost-effectiveness of the FISS program using a €45000 willingness to pay threshold value recommended for Ireland^21^. We assumed the FISS program duration was 52 weeks.

## RESULTS

### Consequences of different FISS reward schedules of a likely implementation scenario in SHCPAs

**Figure 1:**
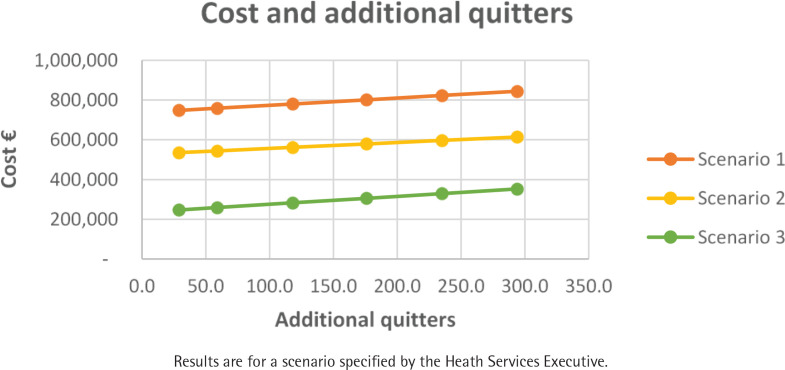
Cost and additional quitters by scenario for a targeted cohort of smokers across Sláintecare Healthy Community Program areas (N=3500)

**Figure 2:**
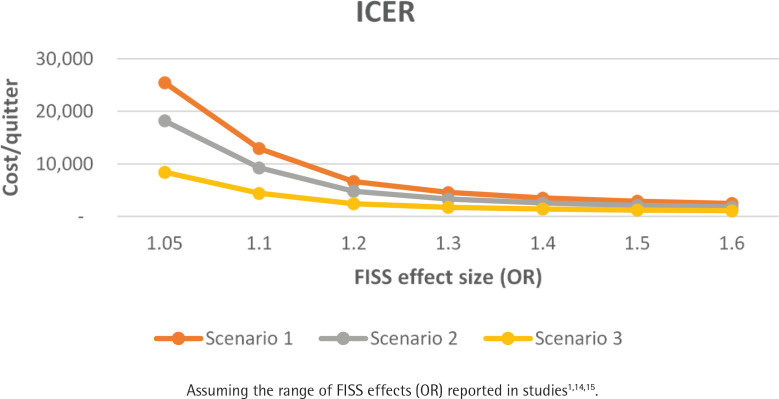
Incremental cost per quitter by differing FISS effect size and scenario for a targeted cohort of smokers across Sláintecare Healthy Community Program areas (N=3500)

Table 2 summarizes the consequences of the reward schedule expressed in terms of the number of additional quitters, budget impact, and the ICER for a cohort of 3500 smokers across SHCPAs. The number of additional quitters did not change across each reward schedule scenario (depends on effect size) (Figure 1). FISS payment schedule as per Scenario 1 is most costly, and Scenario 3 is the least costly. FISS appears to be more cost-effective with rewards as specified in Scenario 3 (Figures 1 and 2).

**Table 2:** Summary of FISS consequences by reward scenario for a cohort of smokers (N=3500)

*Effect size*	*Number of additional quitters*	*Budget impact (€)*	*ICER (cost/quitter) (€)*
**Scenario 1**			
1.05	29	748212	25472
1.1	59	758841	12917
1.2	118	780100	6639
1.3	176	801358	4547
1.4	235	822616	3501
1.5	294	843875	2873
1.6	353	865133	2454
**Scenario 2**			
1.05	29	535058	18215
1.1	59	543870	9258
1.2	118	561494	4779
1.3	176	579119	3286
1.4	235	596743	2539
1.5	294	614368	2092
1.6	353	631992	1793
**Scenario 3**			
1.05	29	246743	8400
1.1	59	258493	4400
1.2	118	281992	2400
1.3	176	305492	1733
1.4	235	328991	1400
1.5	294	352491	1200
1.6	353	375990	1067

ICER: incremental cost-effectiveness ratio. Assuming the range of FISS effects (OR) reported in studies^1,14,15^.

### Consequences of the cost-effectiveness trade-off between the incentive payment schedule and effect size

Scenario 2 and Scenario 3 appear to be more cost-effective than Scenario 1. Comparing the budget impact at FISS effect size of 50%, Scenario 2 is 27%, and Scenario 3 is 58% cheaper to implement compared to Scenario 1. However, retention of individuals in the FISS program for 52 weeks is likely to be more successful using the payment schedule of Scenario 1. Regularly scheduled payments are more likely to motivate and incentivize individuals to stay in the program, resulting in higher 12-month quit rates^1^. Scenario 1 is not the most cost-effective option, with an ICER of €2873 per successful quitter at a FISS effect size of 50%. Using Scenario 2 payment schedules to achieve the same ICER would only be feasible at a much lower FISS effect size of 30–35%, resulting in fewer successful quitters. Similarly, a much smaller effect size of 10–15% using Scenario 3 scheduled payments would provide the same ICER of €2873 but for an even lower number of successful quitters. Consequently, despite all scenarios being cost-effective, it is important to consider the trade-offs in terms of the expected effect size and the potential number of successful quitters.

### Consequences of different FISS payment schedules at the population level

Assuming the FISS program is rolled out nationally for the proportion of the smoking population (n=20000) using the HSE stop smoking support program (i.e. telephone and face-to-face services), a substantial additional reward payment would be required. To achieve quit success rates at a FISS size of 50%, the required budget would increase to €4.8 million for Scenario 1, €3.5 million for Scenario 2, and €2.0 million for Scenario 3. The scale of the implementation of the FISS program would significantly impact the required budget for roll-out.

### Consequences of FISS by sociodemographic characteristics

**Figure 3:**
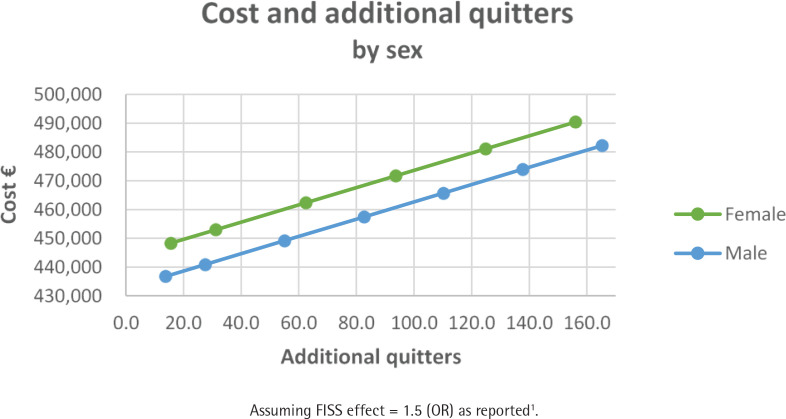
Cost and additional quitters by sex assuming a FISS effect size of 50%

**Figure 4:**
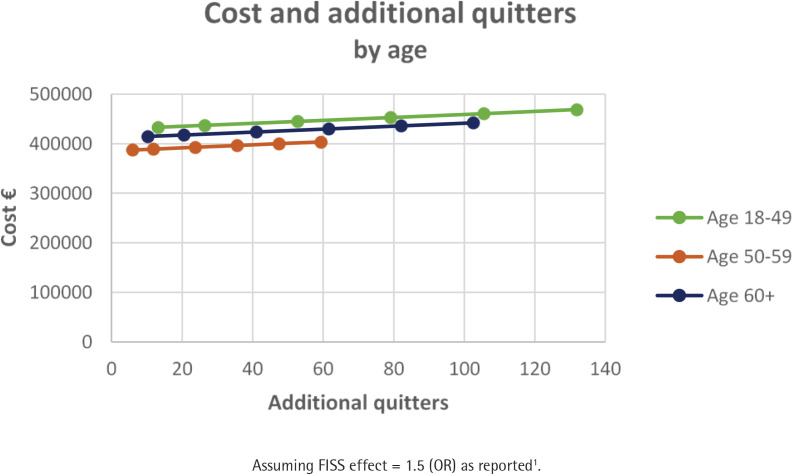
Cost and additional quitters by age assuming a FISS effect size of 50%

**Figure 5:**
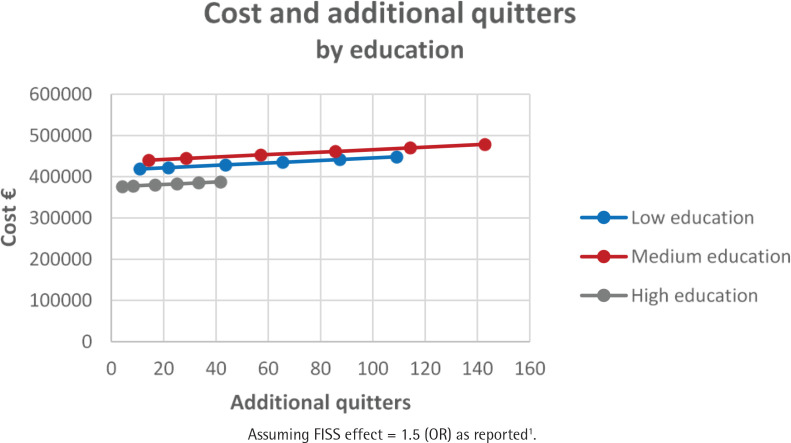
Cost and additional quitters by education level assuming a FISS effect size of 50%

Table 3 summarizes the consequences of FISS as per Scenario 3 for a cohort of 3500 smokers and assuming a FISS effect size of 50%. The smoking quit rates across these groups are reported in Supplementary file Tables S1–S3. FISS is more cost-effective for females by a factor of 1.1 compared to males (Figure 3). The youngest age group is more cost-effective by a factor of 1.9 compared to the oldest age group (Figure 4). Those with a medium level of education are more cost-effective by a factor of 2.7 compared to those with a low level of education (Figure 5). This clearly shows that the FISS program has differing consequences across different sociodemographic characteristics of smoking individuals. However, these estimates are based on explicit assumptions of variation in effect size for different groups of smokers based on data from the existing smoking cessation support program without financial rewards.

**Table 3:** Summary of FISS consequences by sociodemographic characteristics for a specific SHCPA cohort of smokers enrolled in the FISS program (N=3500)

*Characteristics*	*Number of additional quitters*	*Budget impact (€)*	*ICER (cost/quitter) (€)*
**Sex**			
Female	156	490425	3143
Male	138	473943	3441
**Age** (years)			
18–49	132	468669	3554
50–59	59	403401	6799
≥60	103	442298	4313
**Education level**			
Low	109	448231	4107
Medium	143	478558	3350
High	42	387578	9282

ICER: incremental cost-effectiveness ratio. Assuming FISS effect = 1.5 (OR) as reported^1^.

## DISCUSSION

This analysis has identified important challenges decision-makers face when designing the reward structure of FISS programs. The design of the reward schedule has clear trade-offs between the size of the reward and the retention of individuals. However, little empirical evidence is available to demonstrate this relationship. We determined that a FISS program if implemented for a cohort of 3500 smokers across SHCPAs in Ireland, can result in a budget impact in the range €250000 – €870000. To inform decision-makers, we also illustrated the consequences of the cost-effectiveness trade-off between different payment schedules and the expected effect size of the FISS program. It would appear that all scenarios, with even just a modest effect size, are cost-effective at the threshold value of €45000 per quitter. Our findings are similar to those of previous studies, which have also identified FISS programs as cost-effective across different smoking populations^14,16,22,23^. While most of this evidence was based on experimental data from randomized controlled trials, the findings were limited to small population samples. In contrast, our analysis considered the population-level consequences, which may better inform policy and decision-makers of the potential benefits of FISS programs at the national level. In this regard, we estimated that implementing a FISS program at the national level for approximately 20000 smokers in Ireland would cost between €2.0 million and €4.8 million, subject to the chosen reward schedule.

Additionally, we have illustrated that decision-makers should consider the equity issues across different sociodemographic groups, as some groups would be more likely to take part in FISS programs and may benefit more from smoking cessation support. We illustrate how the effects might differ by sociodemographic characteristics and, as a result, how the retention rate and successful quit rates will differ. This is in line with recent evidence suggesting that smoking cessation rates are greater among females, older individuals, and those with the highest levels of education^24-26^. This would suggest that FISS programs if targeted across certain sociodemographic groups, could achieve the highest long-term smoking cessation rates.

### Limitations

This study has several limitations. First, due to lacking experimental data, e.g. from randomized controlled trials within the Irish context, our analysis was limited to using data on smoking quit rates and drawing explicit assumptions of variation in effect size from publicly available sources on existing smoking cessation support programs that do not offer financial incentives^10^. As a result, this limited our analysis to focus on immediate cost-effectiveness results, which may have overlooked the long-term healthcare savings from reduced smoking-related morbidity and mortality. Evidently, an extended economic evaluation accounting for lifetime costs and quality-adjusted life years (QALYs) could offer a complete assessment of the financial benefits of the FISS program. The current study focuses on modeling the various financial incentive scenarios based on various assumptions informed by the Cochrane review^1^ and is, therefore, not a cost-effectiveness study embedded in a randomized controlled trial nor an implementation study.

Second, our different scenario specifications were primarily informed by other published literature^1,4^. As a result, our assumptions of a linear relationship between reward sizes and smoking quit rates may have oversimplified and potentially underestimated the real-world settings and impact of the FISS program. However, our basis for assuming a linear relationship stemmed from a systematic review and meta-analysis of randomized controlled trials, many of which also assumed this relationship^1^. Indeed, behavioral responses to incentives can be nonlinear and influenced by various psychosocial factors^5,27^. Additionally, other factors such as smokers’ motivation at the time of enrolment, support systems, and the use of other substances were not considered in our analysis as potential confounding variables that could have influenced smoking cessation success and response to FISS. Therefore, our analysis was simplified due to the overall inconclusive evidence of this relationship^1,4^ and limited Irish data on other factors, such as behavioral responses to incentives at the population level. Our study illustrates that these factors could impact cost-effectiveness. Future studies should account for such factors, with further exploration of the relationship between financial incentive size and timing, in order to improve and inform the more appropriate FISS structures.

### Implications

This study aimed to provide important evidence to decision-makers and policymakers when designing FISS programs. However, potential barriers to implementing FISS programs among stakeholders in Ireland should also be considered. Despite the Irish general public’s acceptability of implementing FISS^28^, a similar study exploring stakeholders’ acceptability in terms of potential resistance and logical challenges would further enrich this study’s applicability at the national level. One example is the COMPASS project in Ireland^29^, an implementation study that will be informed by the analysis reported in this study and which will specifically focus on identifying the enablers and barriers of FISS implementation in Ireland using mixed-methods research.

## CONCLUSIONS

This study highlights the importance of considering different FISS schedules and potential quit effects when designing tobacco cessation programs. We also highlight that FISS programs targeted at certain sociodemographic groups could potentially achieve the highest long-term smoking cessation rates. Additionally, we identified some of the important challenges decision-makers face when designing the reward structure of FISS programs. The acceptability of the FISS structures may differ among stakeholders and should be explored.

## Supplementary Material



## Data Availability

The data supporting this research are available from the authors on reasonable request.
