# Supplementary material for: Financial incentives to stop smoking: Potential financial consequences of different reward schedules
Source: Tob Prev Cessat. 2024 Jul 12;10:10.18332/tpc/190617. doi: 10.18332/tpc/190617 (PMC11241474; doi:10.18332/tpc/190617)
Supplement: Supplementary file 1 [file TPC-10-30-s1.pdf]

Table S1. Age-specific smoking quit rates for a cohort of 3,500 individuals obtained from QuitManager data 2021-2022

Age group	%
**Enter programme**	
18-49 years	48.8%
50-59 years	22.0%
60-older	29.2%
**4 wks smoke free**	
18-49 years	25.2%
50-59 years	11.1%
60-older	16.8%
**12 wks smoke free**	
18-49 years	17.1%
50-59 years	7.5%
60-older	12.2%
**52 wks smoke free**	
18-49 years	7.5%
50-59 years	3.4%
60-older	5.9%

Table S2. Sex-specific smoking quit rates for a cohort of 3,500 individuals obtained from QuitManager data 2021-2022

Sex	%
**Enter programme**	
Female	55.6%
Male	44.4%
**4 wks smoke free**	
Female	29.4%
Male	23.7%
**12 wks smoke free**	
Female	20.2%
Male	16.5%
**52 wks smoke free**	
Female	8.9%
Male	7.9%

Table S3. Education-specific smoking quit rates for a cohort of 3,500 individuals obtained from QuitManager data 2021-2022

Education level	%
**Enter programme**	
Low education	36.1%
Medium education	46.4%
High education	17.6%
**4 wks smoke free**	
Low education	18.1%
Medium education	25.9%
High education	9.1%
**12 wks smoke free**	
Low education	12.0%
Medium education	18.9%
High education	5.8%
**52 wks smoke free**	
Low education	6.2%
Medium education	8.2%
High education	2.4%

© 2024 Valentelyte G. et al.
